# Supplementary material for: Unraveling two distinct polymorph transition mechanisms in one n-type single crystal for dynamic electronics
Source: Nat Commun. 2023 Mar 21;14:1304. doi: 10.1038/s41467-023-36871-9 (PMC10030468; doi:10.1038/s41467-023-36871-9)
Supplement: Supplementary file 1 — Supplementary Information [file 41467_2023_36871_MOESM1_ESM.pdf]

Supplementary Information

Unraveling Two Distinct Polymorph Transition Mechanisms in One n-Type Single Crystal for Dynamic Electronics

Daniel William Davies[1]&, Bumjoon Seo[2]&, Sang Kyu Park[1], Stephen B. Shiring[2], Hyunjoong Chung[1], Prapti Kafle[1], Dafei Yuan[3], Joseph W. Strzalka[4], Ralph Weber[5], Xiaozhang Zhu[3], Brett M. Savoie*[2], Ying Diao*[1,6]

[1]Department of Chemical and Biomolecular Engineering, University of Illinois at Urbana-Champaign, 600 South Mathews Avenue, Urbana, IL 61801, USA.

[2]Davidson School of Chemical Engineering, Purdue University, 480 W Stadium Ave, West Lafayette, IN 47907

[3]Beijing National Laboratory for Molecular Sciences, CAS Key Laboratory of Organic Solids, Institute of Chemistry, Chinese Academy of Sciences, Beijing 100190, P. R. China

[4]X-Ray Science Division, Argonne National Laboratory, Argonne, IL 60439, USA

[5]Bruker BioSpin Corp., 15 Fortune Drive, Billerica, MA 01821, United States

[6]Beckman Institute for Advanced Science and Technology 405 N. Mathews Ave. M/C 251, Urbana, IL 61801

Note: current address of Dr. Sang Kyu Park – Institute of Advanced Composite Materials, Korea Institute of Science and Technology, Joellabuk-do, 55324, South Korea

Current address of Dr. Dafei Yuan – College of Materials Science and Engineering, Hunan University, Changsha, 410082, China

Current address of Dr. Bumjoon Seo – Department of Chemical and Biomolecular Engineering, Seoul National University of Science and Technology, 232 Gongneung-ro, Nowon-gu, Seoul 01811, Republic of Korea

& These authors contributed equally to this work

For correspondence: yingdiao@illinois.edu, bsavoie@purdue.edu

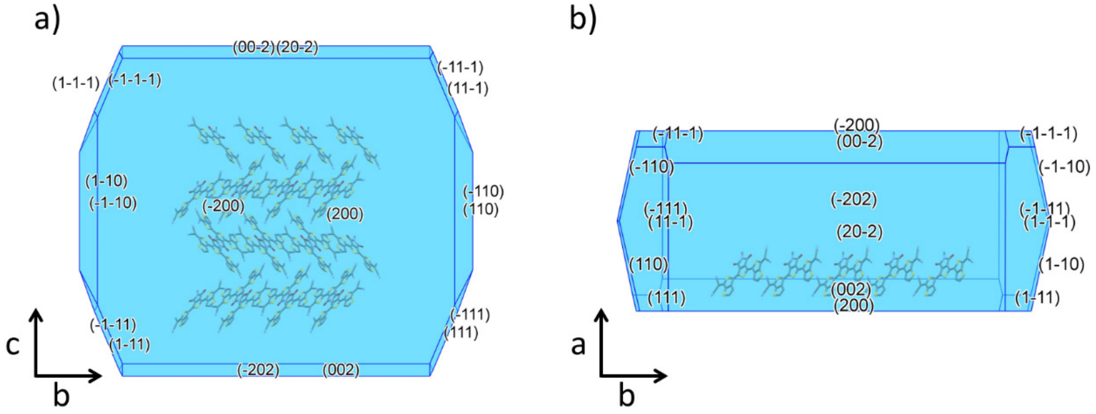

Supplementary fig. 1. BFDH Calculations. Visualization of the BFDH morphology to approximate an expected shape of the crystals based on using the experimental polymorph I structure. The calculations were performed in mercury and look down (a) *a** and (b) *c*-direction. The calculations suggest the largest plane should be the (200) plane, growing along the 1D π-stacks, consistent with the crystal shapes observed from dropcasting.

Supplementary Table 1. Statistics for the I-II transition in single crystals

Crystal	Cooling Transition Temperature (°C)	Calibrated Cooling Transition Temperature (°C)	Heating Transition Temperature (°C)	Calibrated Heating Transition Temperature (°C)	Hysteresis (°C)	Midpoint Temp (°C)	Original length (μm)	Heated Length (μm)	% change in length
090117-1	173.9	156.5	185.8	166.9	11.9	161.7	107.4	104.9	-2.35
090117-2	172.9	155.6	191.4	171.7	18.2	163.7	143.8	140.1	-2.60
090117-3	171.7	154.5	183.6	164.9	11.9	159.7	133.6	129.1	-3.38
090117-4	169.5	152.6	184.8	166.0	15.3	159.3	143.1	141.7	-0.96
091217-2	165.8	149.4	191.9	172.2	26.1	160.8	103.3	100.8	-2.50
110217-1	165.1	148.8	185.7	166.8	20.6	157.8	368.7	358.7	-2.73
110217-2	166.5	150.0	182.4	163.9	15.9	156.9	231.9	221.4	-4.51
110917-4-1	171.8	154.6	190.5	171.0	18.7	162.8	229.4	225.1	-1.88
110917-4-3	172	154.8	187	167.9	15	161.4	228.1	220.8	-3.19
110917-4-4	173.3	155.9	190	170.5	16.7	163.2	210.8	201.1	-4.58
110917-4-5	174.3	156.8	190.7	171.1	16.4	164.0	212.5	205.0	-3.56
110917-5-1	169.3	152.4	179	160.9	9.7	156.7	224.8	219.7	-2.26
110917-5-2	171	153.9	189	169.7	18	161.8	390.6	381.7	-2.28
110917-6.1-1	166.8	150.3	186.1	167.1	19.3	158.7	160.4	151.7	-5.43
110917-6.1-2	174.8	157.2	187.1	168.0	12.3	162.6	258.0	250.1	-3.06
110917-6-1	164.8	148.5	180.1	161.9	15.3	155.2	265.2	251.3	-5.23
110917-6-2	161.1	145.3	182.9	164.3	21.8	154.8	281.2	264.3	-6.03
110917-6-3	162.9	146.9	173.1	155.8	10.2	151.3	166.0	157.8	-4.92
110917-6-4	163.6	147.5	179.6	161.4	16	154.5	292.2	276.9	-5.23
120117-PtS-1-1	175	157.4	185.7	166.8	10.7	162.1	309.4	292.6	-5.44
120117-PtS-1-2	169.1	152.3	192.8	173.0	23.7	162.6	335.3	321.1	-4.23
120117-PtS-1-3	166	149.6	188.3	169.0	22.3	159.3	304.7	294.6	-3.33
120117-PtS-2-1	168.5	151.7	194.1	174.1	25.6	162.9	365.3	353.0	-3.37
120117-PtS-2-2	172.8	155.5	181.7	163.3	8.9	159.4	256.3	250.8	-2.13
120117-PtS-2-3	175.6	157.9	189	169.7	13.4	163.8	117.0	112.4	-3.98
120517-2-1	167.2	150.6	192.4	172.6	25.2	161.6	341.6	330.2	-3.33
120517-2-2	168.2	151.5	182.4	163.9	14.2	157.7	258.0	248.9	-3.53
120517-2-3	166.6	150.1	179.8	161.6	13.2	155.8	214.1	204.3	-4.60
				**Average**	**16.7**	**160**		**Average**	**-3.59**
				**Standard Deviation**	**5.0**	**3.36**		**Standard Deviation**	**1.28**

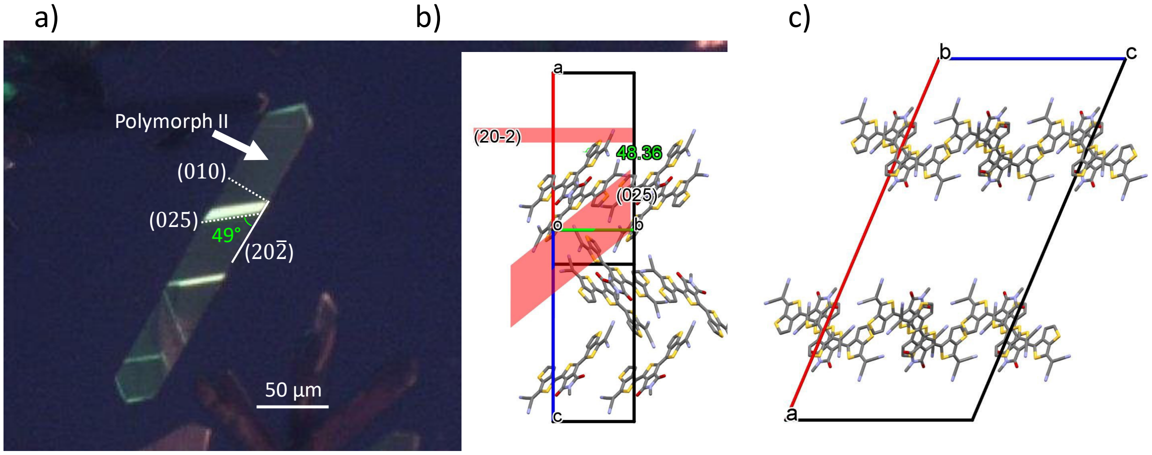

Supplementary fig. 2. **Crystallographic relationship between polymorphs I and II**. (a) POM images of the II-I transition showing both the (025) plane orientation for the phase boundary. (b) The relationship between the (025) plane and the π-stacking plane of the molecule. The angles between the (025) and $(20\bar{2})$ in the POM image (a) was measured using ImageJ. The angle based on the unit cell was measured using Mercury. Angles are indicated in green and show a close correlation between the π-stacking plane and the phase boundary. (c) The (010) looking down the π-stacking columns.

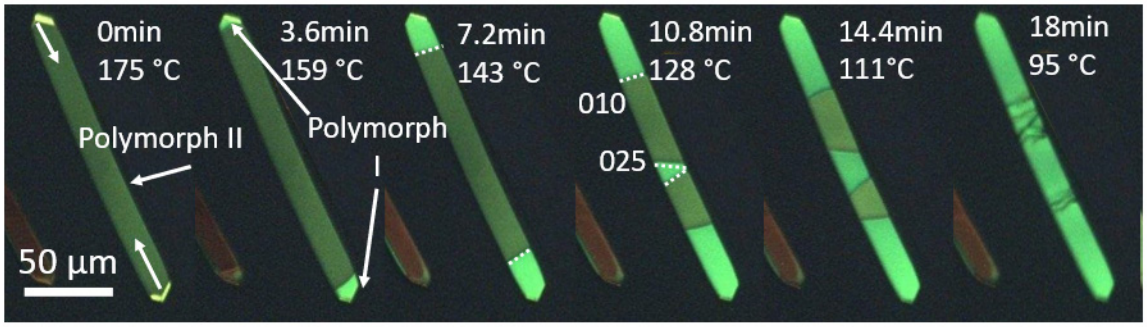

Supplementary fig. 3. **Domain orientation and crystal cracking**. POM images of the II-I transition showing cracking upon convergence of independently initiated phase boundaries. The full movie is shown in Supplementary Movie 5.

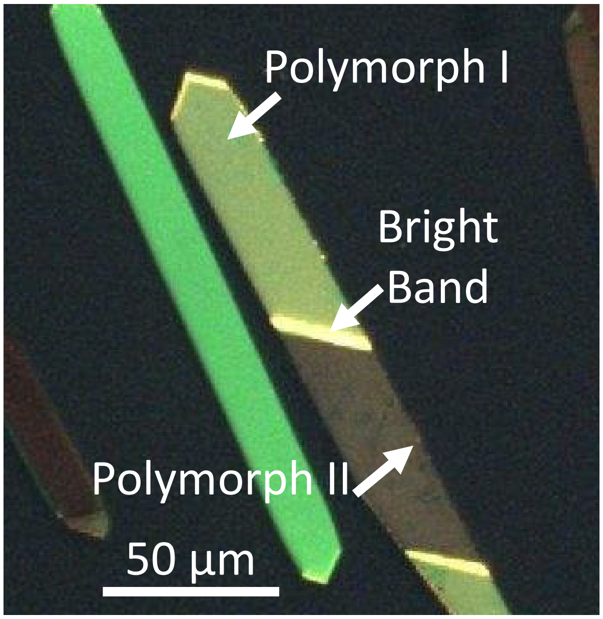

Supplementary fig. 4. **Bright Bands** During the avalanche behavior, bright bands sometimes occur that the phase boundary, suggesting strain buildup along the boundary.

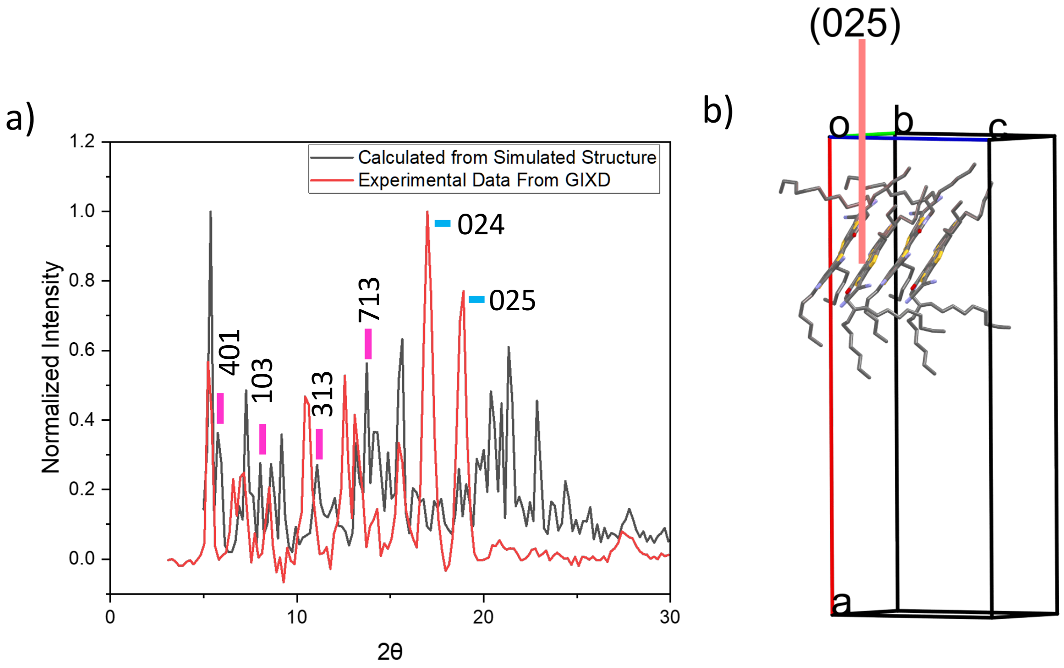

Supplementary fig. 5. Polymorph II simulation. (a) Comparison of powder X-ray diffraction extracted from GIXD data from polymorph II with the simulated pattern from the simulated structure. (b) packing in polymorph II showing the (025) plane aligning with the π-stacking direction in the simulated structure predicted from the GIXD pattern. While the main peaks from the powder diffraction match well, due to the simulation imposing the extracted polymorph II unit cell, we note a few exceptions. The expected diffraction pattern was calculated using Mercury using the simulated structure and a P1 space group due to a lack of certainty on systematic absences in the GIXD pattern. However, the peaks labeled in magenta were expected to be present but appear to not be present and may be due to such systematic absences. On the other hand, for the peaks labeled in blue, we see much higher intensities in the experimental data than predicted from the calculated spectra. This is likely due to extracting the spectra from the GIXD data and these particular peaks occurring at the horizon of the diffraction pattern.

DFT calculations for the polymorph II structure

The polymorphic transition calculations were achieved by relaxing the fractional atomic positions within the initial unit cell of polymorph I (a=51.8 Å, b=10.2 Å, c=24.2 Å, and β=113°) at the new unit cell with the constrained lattice parameters of polymorph II (a=51.6 Å, b=9.90 Å, c=24.5 Å, and β=91.3°) obtained from the SCXRD. The transition was performed at the semi-empirical GFN2-xTB[13] level under periodic boundary conditions as implemented in DFTB+ version 21.2[14]. The transition was comprised of five steps, where the changes in all lattice parameters per step were one-fifth of the overall changes in each lattice parameter. The same procedure was performed on polymorph III (a=b=20.5 Å, c=24.5 Å, and β=90°) starting from the predicted unit cell of polymorph II, but due to the mismatch in the space group between polymorph II and III, a surrogate monoclinic structure (a=24.5 Å, b=20.5 Å, c = 35.5 Å, and β=90°) was used that corresponds to the unit cell of polymorph III under the redefinition of the unit cell with the hexagonal symmetry.

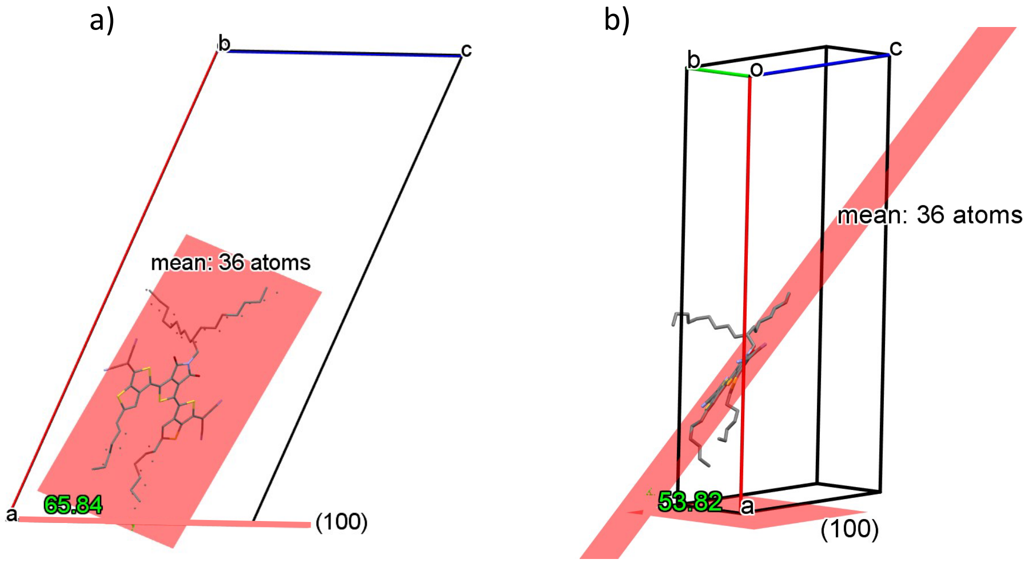

Supplementary fig. 6 Molecular Tilt. The angle between the conjugated core and the (100) plane in (a) polymorph I measured as 66° and (b) polymorph II measured to be 54°.

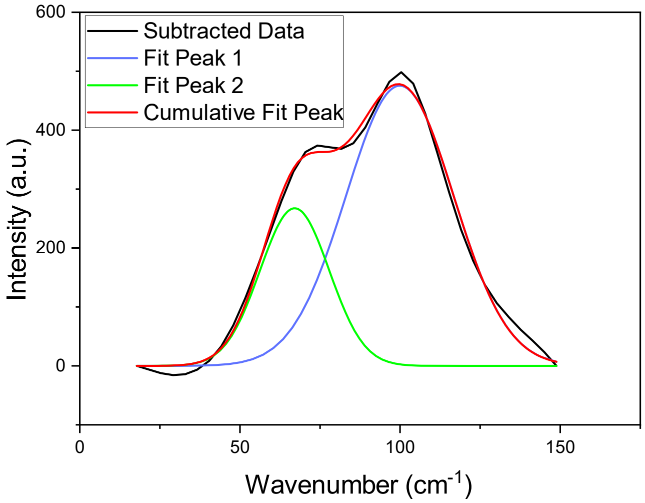

Supplementary fig. 7 Phonon mode fit. Deconvolution of the two phonon modes observed at low wavenumbers during Raman spectroscopy.

Quinoidal Raman Peak Assignment

To interpret changes in the intramolecular vibration region (900-1800 cm^{-1}), we assigned the Raman peaks based on a combination of literature and DFT calculations, as discussed in the methods. DFT simulations of the 2DQTT-o-B vibrational spectra shows one dominant vibrational mode which we expect corresponds to the most intense peak in the experimental Raman spectra (Supplementary fig. 8a,b). This suggests a 200 cm^{-1} systematic shift between the experimental and simulated data, which we attributed to performing the simulations on single molecules. The most intense peak measured at 1387 cm^{-1} experimentally (peak 1 in Supplementary fig. 8b), has typically been assigned the collective vibrations of the C-C/C=C bonds in the conjugated core, known as the effective conjugation coordinate (ECC) mode in similar molecules[1–3]. In simulations of 2DQTT-o-B, we observed the ECC mode (1387 cm^{-1}) comprises 2 peaks exhibiting stretching of the TBT and TPD moieties and stretching along the backbone at 1581 and 1584 cm^{-1} (Supplementary fig. 8c, Supplementary movies 8-9) respectively. Taken together, these vibrational modes stretch along the conjugated C=C bonds, as expected for the ECC mode, and are collectively denoted as $\nu(C{=}C)_{ECC,Q}$ shown in Figure 3e (where Q specifies the quinoidal form). Notably in the experimental spectra, we observe medium intensity peaks at 1409 cm^{-1}, 1522 cm^{-1}, and 1772 cm^{-1} (peaks 2-4 in Supplementary fig. 8b) that seem to correspond to low intensity peaks in the simulated spectra. Discrepancy in simulated spectra, as we explain below, are likely related to effects from the alkyl chains (omitted from simulation) and molecular packing which were prohibitively difficult to simulate in this case. We suggest 1522 cm^{-1} and 1772 cm^{-1} correspond to another $\nu(C{=}C)_{TPD}$ in the central TPD ring and ν(C=O) respectively which have corresponding weak vibrational modes in the simulated spectra at 1636cm^{-1} (Supplementary fig. 8e, Supplementary movie 10) and 1878 cm^{-1} (Supplementary fig. 8f, Supplementary movie 11). While the simulated intensities were much lower than the measured Raman activity, electron-phonon coupling in the solid state and reduction of the molecular symmetry has a dramatic effect on the calculated intensities for these vibrational modes[4–6].

As for the peak at 1409 cm^{-1}, while it seems to correspond to the simulated band at 1610 cm^{-1} (Supplementary fig. 8d, Supplementary movie 12), it also overlaps with the CH_2 deformation peak ($\delta(CH_2)$) in the alkyl chains which the simulation does not capture. This vibrational mode involves scissoring of the CH_2 group (Figure 3e) and has been well studied in literature from alkanes and oligothiophenes with tethered alkyl chains[7–10]. However, assigning these modes based solely on the simulated Raman and literature alone is difficult given the possible overlap with core stretching peaks. In order to confirm the attribution of these peaks to the alkyl chains, we measured the Raman spectra of crystals formed using 2DQTT-C_2C_4, containing the same quinoidal core and only differing in the length of the branched alkyl chains (Supplementary fig. 9). We observed the position and relative intensities of peaks assigned to the quinoidal core show no change upon modification of the alkyl chain. On the other hand, the peak at $\delta(CH_2)$ 1409 cm^{-1} showed substantial reduction in the intensity 2DQTT-C_2C_4, confirming the assignment of this peak to the alkyl chains rather than another core C=C stretching mode. Similarly, based on literature[7–10] and comparison with 2DQTT-C_2C_4, we assign peaks at 1025 cm^{-1} and 1063 cm^{-1} to alkyl C-C stretching of the trans ($\nu(C{-}C)_T$) and gauche ($\nu(C{-}C)_G$) conformations respectively (Supplementary fig. 9). In the C_2C_4 Raman spectra, the trans C-C stretching mode showed a substantial difference in peak position and presents a new peak at a slightly lower wavenumber. This peak splitting is likely due to the shorter C_2 alkyl chains no longer containing the CH_2-CH_2 bond present in the other alkyl chains. Given the only difference between these molecules is the side chains, this confirms our assignments of the alkyl chain vibrations.

The assignment of these modes offers a framework with which to understand the observed peak evolution previously discussed.

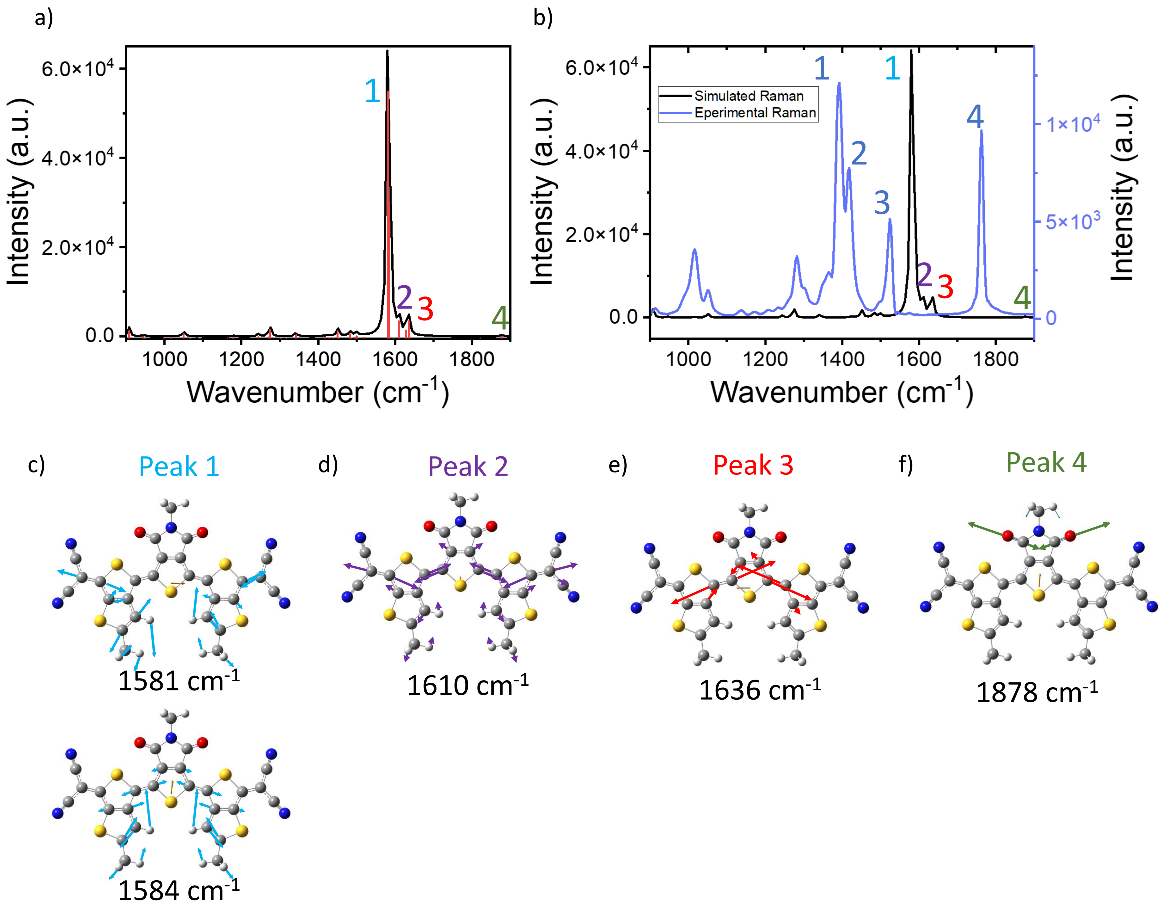

Supplementary fig. 8 Simulated Raman spectra of quinoidal form. (a) Simulated Raman spectroscopy showing 4 major core stretching peaks. (b) Comparison between the simulated (black) and experimental (red) spectroscopy, showing a qualitatively similar fingerprint offset by approximately ~100 cm^{-1}. (c-f) The vibrational modes corresponding to the most Raman active vibrations in each peak corresponding to Supplementary movies 8-12. Based on this, we were able to assign the most intense peak (peak 1) to the ECC mode $\nu(C{=}C)_{ECC,Q}$ Compared to the experimental spectra, the most intense Raman peak was observed near 1387 cm^{-1}, 200 cm^{-1} lower than the simulation results. This is likely due to the simulation being performed on single molecules at low temperatures, and we see this 200 cm^{-1} shift is similar for all the observed peaks.

The most intense simulated peak was calculated to be composed of 2 peaks at 1581 and 1584 cm^{-1} We expect experimental Peak 2 to be mainly the $\delta(CH_2)$ mode based on well documented literature, we could not simulate the full alkyl chains in this simulation. This assignment was confirmed via performing Raman on 2DQTT-C_2C_4 in Supplementary fig. 9. Peaks 3 and 4 we assigned based on simulated weak peaks and

are likely stronger in the experimental spectra as a result of intermolecular interactions in the crystal structure.

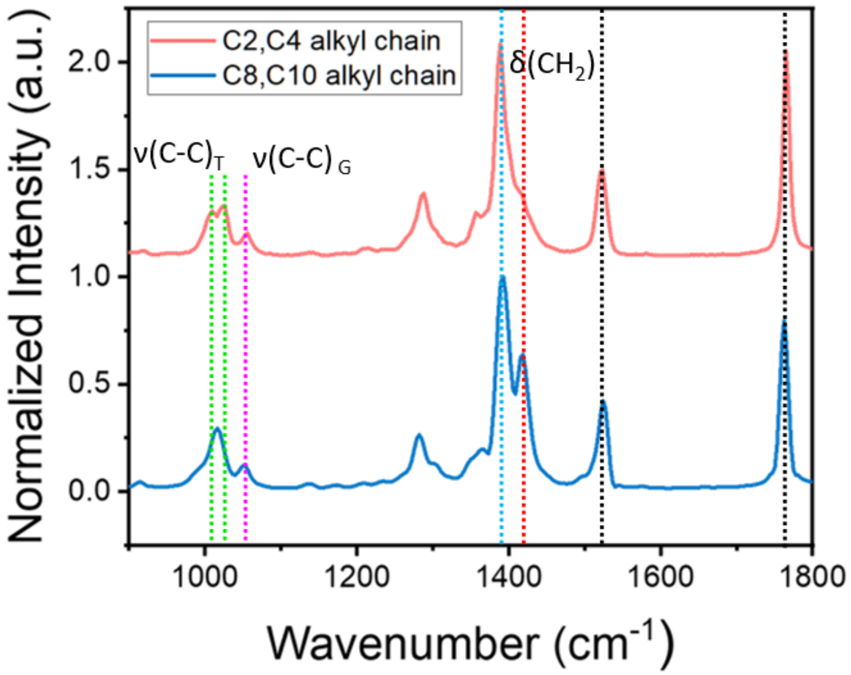

Supplementary fig. 9. **Raman spectra change with side chain modification**. Experimental Raman spectra for 2DQTT-C_8C_{10} (blue) and 2DQTT-C_2C_4 (red) at room temperature in the polymorph I state, showing modulation of the peak splitting of the ν(C-C)$_T$ (green dotted lines) and intensity of the δ(CH_2) vibrational mode associated with the alkyl chains and little to no change to the core C=C stretching peaks (black), confirming these Raman assignments.

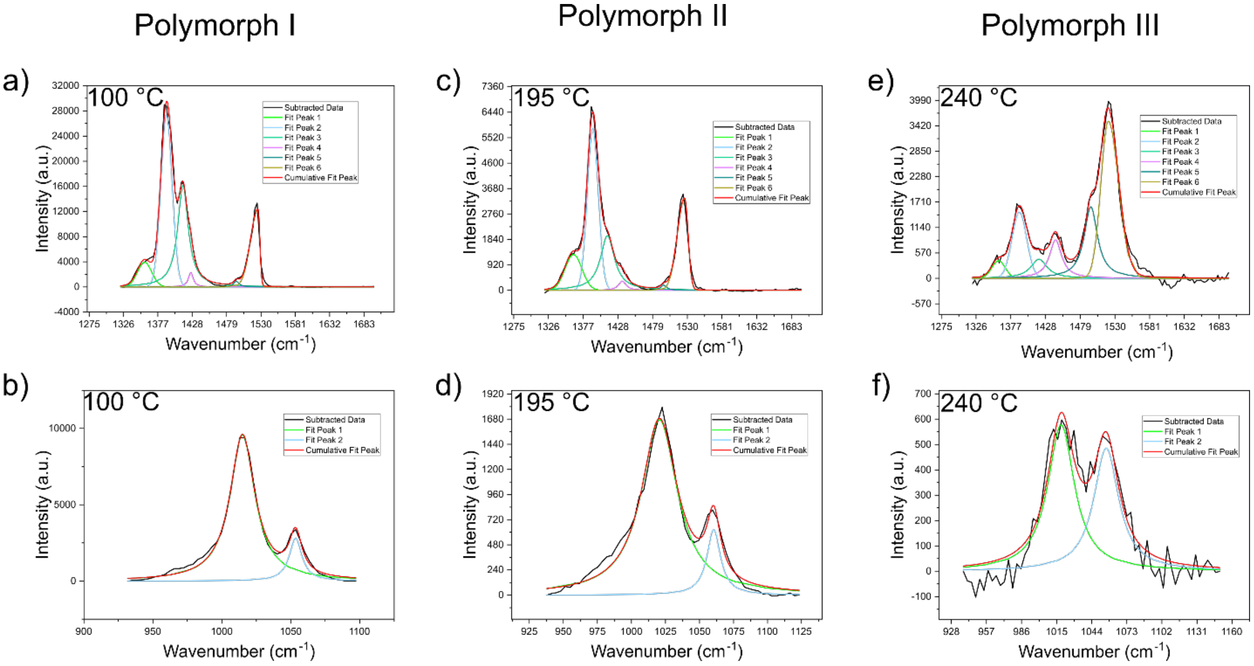

Supplementary fig. 10. **Representative Raman fits for each polymorph.** Representative peak fittings for polymorph I (a,b), polymorph II (c,d), and polymorph III (e,f) in the 1200-1700 cm^{-1} and 900-1100 cm^{-1} regions. Peaks associated with the conjugated core were fit with a gaussian line shape and peaks associated with the alkyl chains were fit with Lorentzian line shapes. All peaks were fitted in each scenario for consistency.

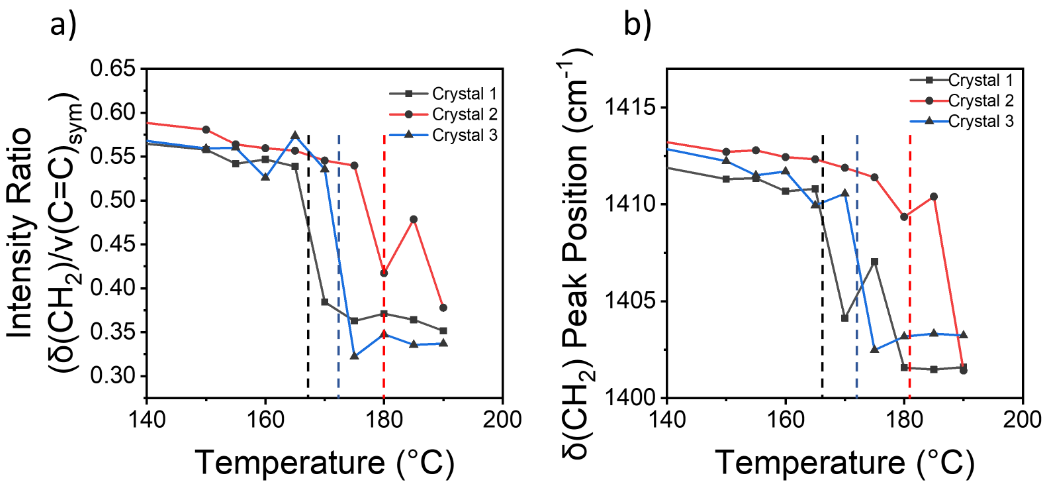

Supplementary fig. 11. Raman spectra changes during I-II transition. Repeated experiments from **Figure 3** showing the consistency of (a) the decrease in the δ(CH_2)/ν(C=C)$_{core}$ intensity ratio and (b) the redshift in the δ(CH_2) peak. This experiment was performed on 3 separate crystals, each of which transitioned at different temperatures indicated by the dashed line. The transition temperatures were observed and recorded based on the shape change evident under the optical microscope linked with the Raman spectroscopy setup. The discrepancy in the data point at 185 °C is likely the result of the avalanche behavior and measuring at different locations in the crystal. This resulted in accidental measurement of polymorph I that had not finished converting when moving the laser back and forth between each crystal.

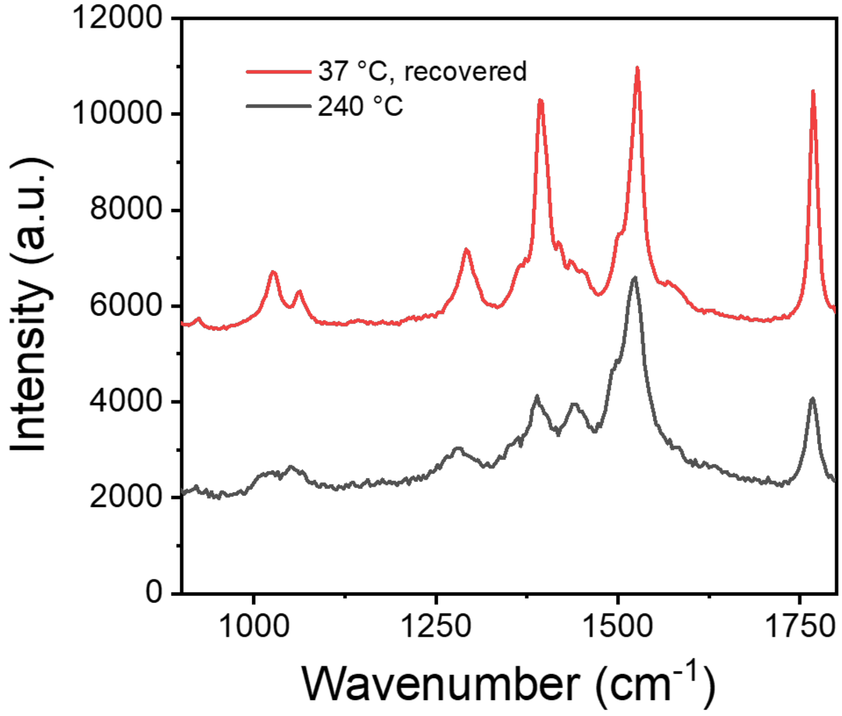

Supplementary fig. 12. Reversibility of polymorph III vibrational changes. Raman spectra of polymorph III at high temperature (black) and after cooling (red). Upon cooling, while the alkyl ν(C-C) stretching has returned to a high ratio, suggesting solidification, biradical signatures are still observed. The intensity has decreased, but the signatures are still observable.

Biradical State DFT Calculations

To investigate this possibility of a quinoidal to aromatic transition, we simulated the vibrational spectra of an aromatic analogue to 2DQTT-o-B by removing the cyano groups and replacing them with hydrogens (Supplementary fig. 10a) which forced the BLA into the aromatic form while still allowing for structural optimization using the ωB97X-D functional and def2TZVPP basis set. From the simulation of the aromatic form of the molecule, we observed 2 main intense peaks which showed a clear symmetric and asymmetric stretching of the core C=C bonds (the aromatic ECC stretching modes, $\nu(C{=}C)_{ECC,A}$, $\nu(C{=}C)_{asym}$), at 1618 and 1635 cm^{-1}, respectively (Supplementary fig. 13b-c, Supplementary movie 14-15). The DFT simulation suggested the aromatic peak should form at higher wavenumbers, approximately a shift of 40 cm^{-1} relative to the quinoidal ECC stretching (Supplementary fig. 13d). This simulated shift is quite close to the experimental difference between the quinoidal ($\nu(C{=}C)_{ECC,Q}$) and aromatic ($\nu(C{=}C)_{ECC,A}$) peaks, which was measured to be 52 cm^{-1}. This suggests the simulated $\nu(C{=}C)_{ECC,A}$ and $\nu(C{=}C)_{asym}$ peaks correspond to the experimentally observed peaks forming at 1440 and 1497 cm^{-1} during the II-III transition (Figure 4f, Supplementary fig. 13e). Moreover, this assignment is consistent with observations in previous literature results[4,11,12], which also showed the aromatic peaks forming at higher wavenumbers compared to the quinoidal vibrational mode.

The broken-symmetry calculations were performed at the B3LYP[15,16]/ 6-31G(d,p)[17,18] level of theory using Gaussian16 Rev. B[19]. The initial geometry of the molecule was taken from the experimental crystal structure of polymorph I, with the alkyl side-chains replaced by methyl groups and the chain ends terminated with hydrogen atoms to remove the errors in energy calculation due to conformational effects. First, stable dimer configurations were found by displacing two molecules in the antiparallel configuration at a series of distances (in steps of 1 Å from 2 to 5 Å) and performing geometry optimization at the singlet state. The optimized geometry went through further geometry optimization with the triplet state for both molecules to generate the initial guess for the geometry of the open shell singlet molecule. Finally, unrestricted wavefunctions were used to converge the SCF to the broken symmetry solution of the molecule with an open shell singlet state. The biradical character y_0 was defined as the occupation number of the lowest unoccupied natural orbital[20]. The singlet-triplet energy gap and the effective electron exchange interaction J_{ab} were calculated as

$$\Delta E_{S-T} = (E_{OS} - E_T)\ \frac{\langle S^2 \rangle_T}{\langle S^2 \rangle_T - \langle S^2 \rangle_{OS}}$$

$$J_{ab} = \frac{E_{OS} - E_T}{\langle S^2 \rangle_T - \langle S^2 \rangle_{OS}}$$

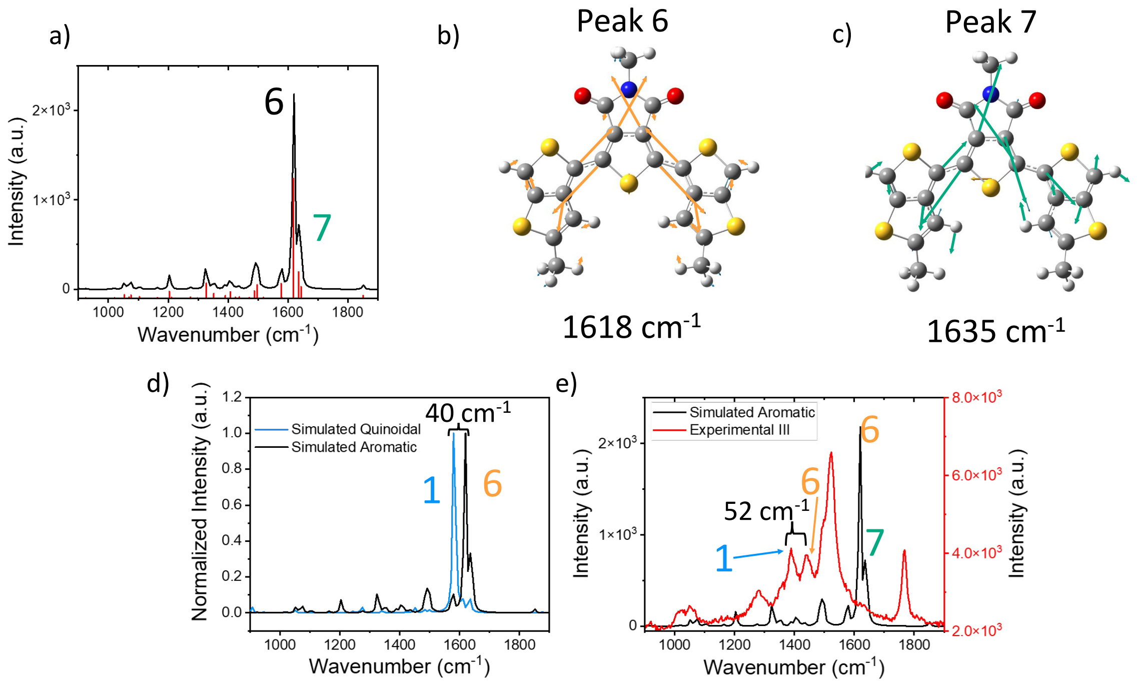

Supplementary fig. 13. Simulated aromatic Raman spectrum (a) Simulated Raman spectroscopy showing 2 major core stretching peaks in the aromatic form of the molecule. The simulation was performed by removing the cyano groups and allowing full relaxation of the molecule. The main vibrational mode is associated with (b) the ECC symmetric mode $\nu(C{=}C)_{ECC,A}$ and (c) the ECC asymmetric mode $\nu(C{=}C)_{ECCasym,A}$, corresponding to Supplementary movies 14-15 respectively. (d) Comparison between the simulated aromatic (black) and quinoidal (blue) Raman spectroscopy, showing an increase of 40 cm^{-1} between the ring breathing modes (most intense peaks). (e) Comparison between the simulated aromatic Raman spectra (black) and the experimental spectra taken from polymorph III at 240 °C. The experimental spectra shows an increase of 52 cm^{-1}, similar to the shift based on the simulated Raman spectra of 40 cm^{-1}, and consistent with trends shown in literature.

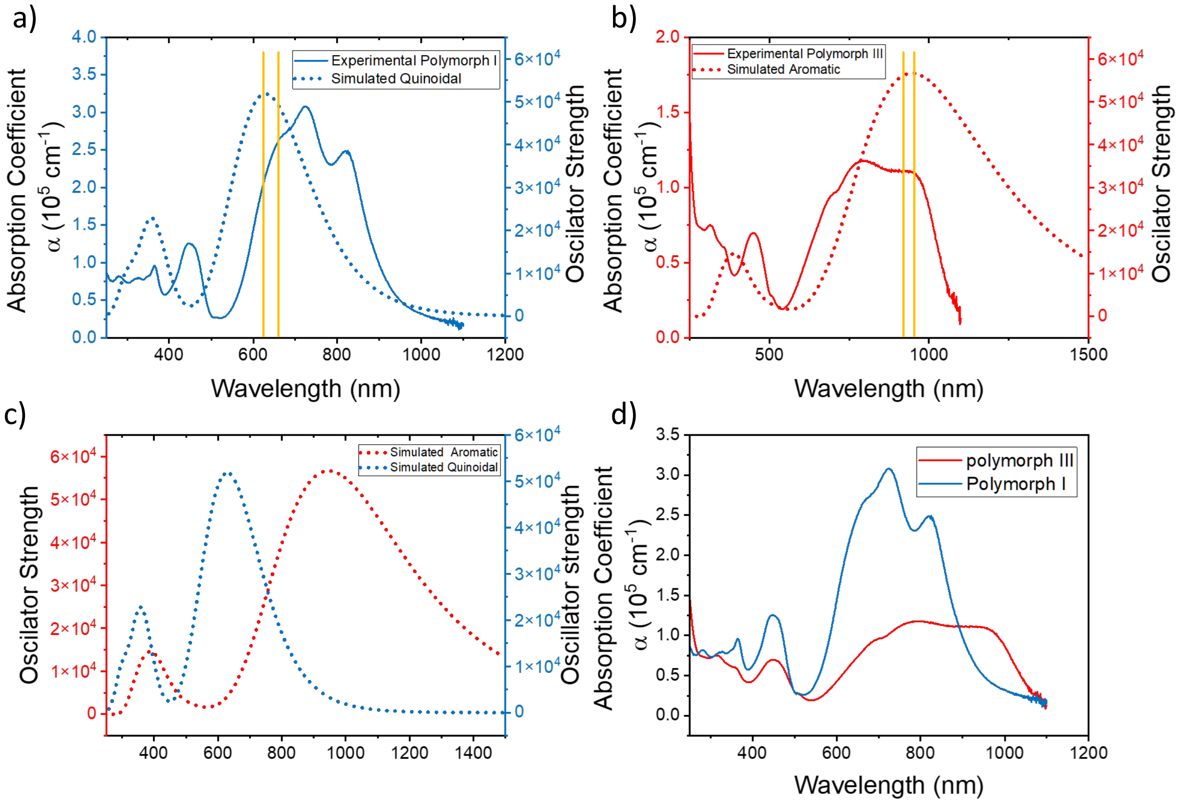

Supplementary fig. 14. **Simulated UV-Vis spectra of quinoidal and aromatic forms**. (a) Comparison of simulated UV-Vis absorption spectra for the *quinoidal* form with polymorph I. (b) comparison of simulated UV-Vis absorption spectra for the *aromatic* form with polymorph III. (c) showing the red shift in the lowest energy peak from the quinoidal to aromatic form and (d) a corresponding redshift in the experimental absorption spectra in polymorphs I and III.

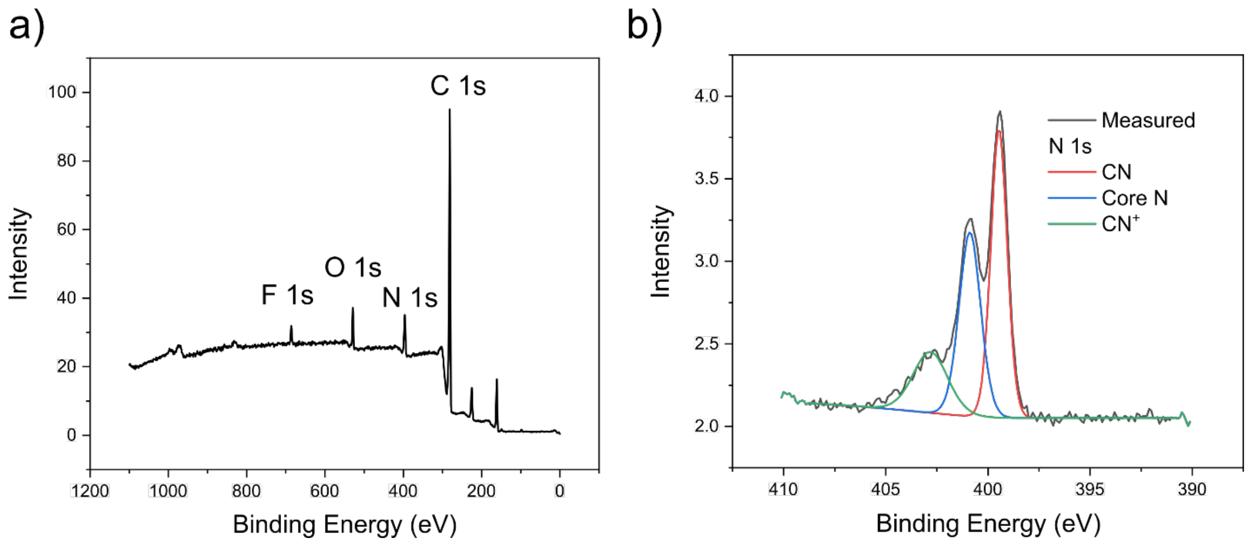

Supplementary fig. 15. XPS spectra of film. (a) Full XPS spectra of a film of 2DQTT-o-B at room temperature. (b) Deconvolution of the three nitrogen peaks assigned to the neutral cyano group nitrogen (red), the core nitrogen (blue) and the charged cyano group nitrogen (green). The area percentage of charged nitrogen relative to the total cyano group nitrogen shows approximately 20% charged N species.

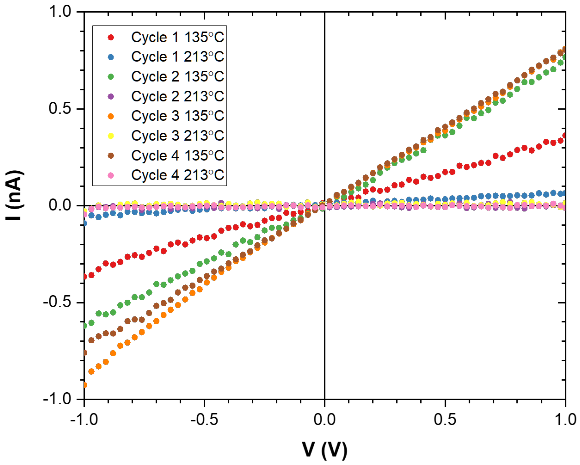

Supplementary fig. 16. Measured actuator devices. Raw data showing the device performance from cycling in Figure 7c showing that in the disconnected state, the measurement reaches the noise floor for detection.

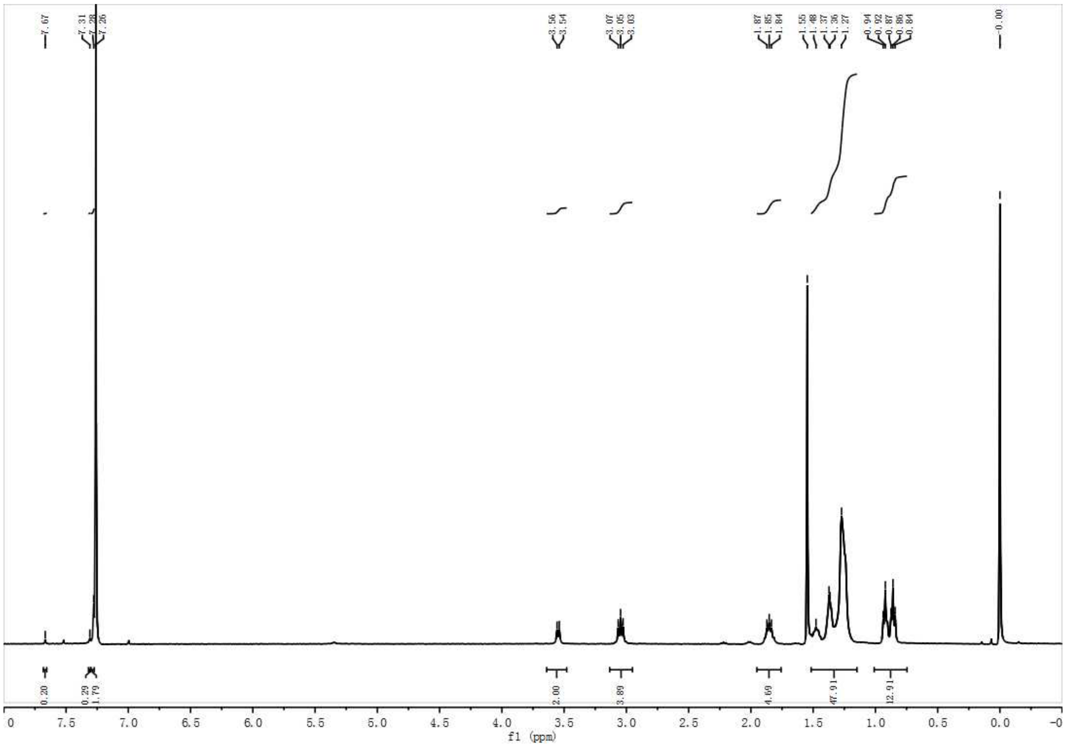

Supplementary fig. 17. ^{1}H NMR of 2DQTT-o-B

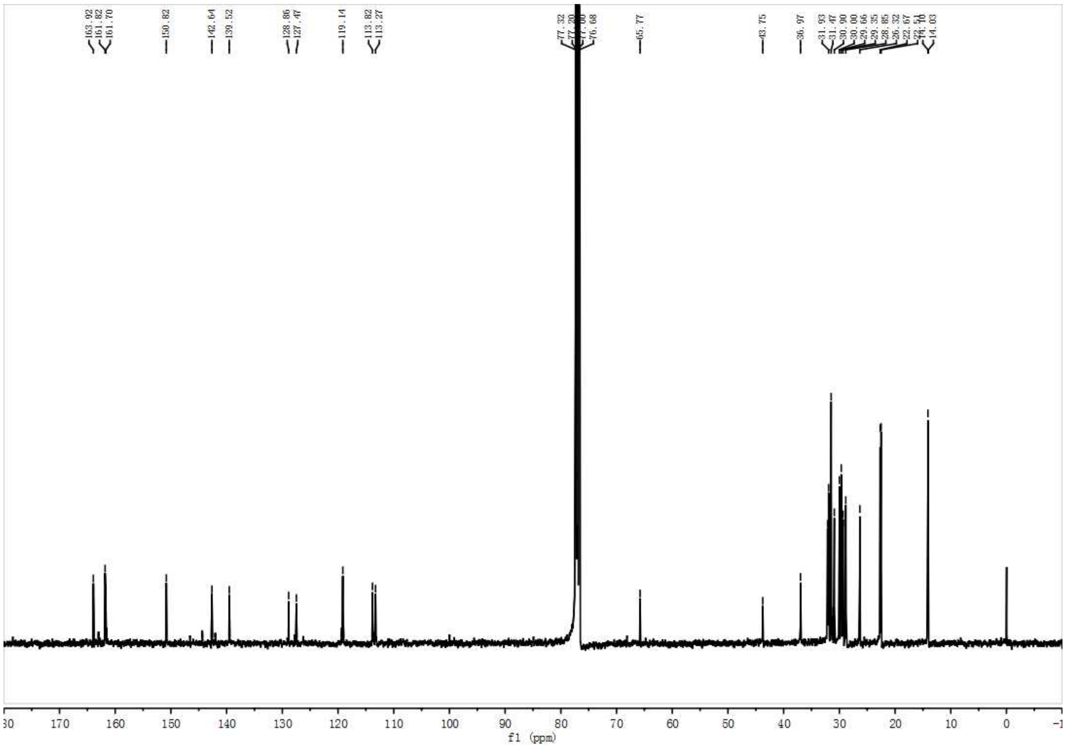

Supplementary fig. 18. ^{13}C NMR of 2DQTT-o-B

Supplementary References

1. Casado, J., Ponce Ortiz, R. & López Navarrete, J. T. Quinoidal oligothiophenes: new properties behind an unconventional electronic structure. *Chemical Society Reviews* **41**, 5672–5686 (2012).
2. Castiglioni, C., Lopez Navarrete, J. T., Zerbi, G. & Gussoni, M. A simple interpretation of the vibrational spectra of undoped, doped and photoexcited polyacetylene: Amplitude mode theory in the GF formalism. *Solid State Communications* **65**, 625–630 (1988).
3. Ferrari, A. C., Robertson, J., Castiglioni, C., Tommasini, M. & Zerbi, G. Raman spectroscopy of polyconjugated molecules and materials: confinement effect in one and two dimensions. *Philosophical Transactions of the Royal Society of London. Series A: Mathematical, Physical and Engineering Sciences* **362**, 2425–2459 (2004).
4. Casado, J., Hernández, V. & Navarrete, J. T. L. Vibrational Raman Shifts and Aromaticity: The Case of Oligothiophenes. *The Chemical Record* **15**, 1110–1118 (2015).
5. Sjöqvist, J. *et al.* A combined MD/QM and experimental exploration of conformational richness in branched oligothiophenes. *Physical Chemistry Chemical Physics* **16**, 24841–24852 (2014).
6. Casado, J., Ortiz, R. P. & Navarrete, J. T. L. Quinoidal oligothiophenes: new properties behind an unconventional electronic structure. *Chem. Soc. Rev.* **41**, 5672–5686 (2012).
7. Venkataraman, N. V., Bhagyalakshmi, S., Vasudevan, S. & Seshadri, R. Conformation and orientation of alkyl chains in the layered organic–inorganic hybrids: (CnH2n+1NH3)2PbI4 (n = 12,16,18). *Physical Chemistry Chemical Physics* **4**, 4533–4538 (2002).
8. G. Brown, K., Bicknell-Brown, Ellen. & Ladjadj, Meriem. Raman-active bands sensitive to motion and conformation at the chain termini and backbones of alkanes and lipids. *The Journal of Physical Chemistry* **91**, 3436–3442 (2002).

9. J. Orendorff, C., W. Ducey, M. & E. Pemberton, J. Quantitative Correlation of Raman Spectral Indicators in Determining Conformational Order in Alkyl Chains. *The Journal of Physical Chemistry A* **106**, 6991–6998 (2002).
10. Luz-Lima, C. *et al.* α-l-Glutamic acid under high pressure: Phase transitions studied by Raman spectroscopy. *Vibrational Spectroscopy* **86**, 343–349 (2016).
11. Yang, K. *et al.* Stable Organic Diradicals Based on Fused Quinoidal Oligothiophene Imides with High Electrical Conductivity. *Journal of the American Chemical Society* **142**, 4329–4340 (2020).
12. Casado, J. & López Navarrete, J. T. The longest quinoidal oligothiophene: A Raman story. *The Chemical Record* **11**, 45–53 (2011).
13. GFN2-xTB—An Accurate and Broadly Parametrized Self-Consistent Tight-Binding Quantum Chemical Method with Multipole Electrostatics and Density-Dependent Dispersion Contributions | Journal of Chemical Theory and Computation. https://pubs.acs.org/doi/10.1021/acs.jctc.8b01176.
14. Hourahine, B. *et al.* DFTB+, a software package for efficient approximate density functional theory based atomistic simulations. *J. Chem. Phys.* **152**, 124101 (2020).
15. Becke, A. D. Density-functional thermochemistry. III. The role of exact exchange. *J. Chem. Phys.* **98**, 5648–5652 (1993).
16. Lee, C., Yang, W. & Parr, R. G. Development of the Colle-Salvetti correlation-energy formula into a functional of the electron density. *Phys. Rev. B* **37**, 785–789 (1988).
17. Hehre, W. J., Ditchfield, R. & Pople, J. A. Self—Consistent Molecular Orbital Methods. XII. Further Extensions of Gaussian—Type Basis Sets for Use in Molecular Orbital Studies of Organic Molecules. *J. Chem. Phys.* **56**, 2257–2261 (1972).
18. Francl, M. M. *et al.* Self-consistent molecular orbital methods. XXIII. A polarization-type basis set for second-row elements. *J. Chem. Phys.* **77**, 3654–3665 (1982).
19. Frisch, M. J. *et al.* Gaussian 16 Rev. C.01. (2016).

20. Kishi, R. *et al.* Diradical Character Tuning for the Third-Order Nonlinear Optical Properties of Quinoidal Oligothiophenes by Introducing Thiophene-S,S-dioxide Rings. *Chemistry – A European Journal* **22**, 1493–1500 (2016).
