# Supplementary material for: Unraveling two distinct polymorph transition mechanisms in one n-type single crystal for dynamic electronics
Source: Nat Commun. 2023 Mar 21;14:1304. doi: 10.1038/s41467-023-36871-9 (PMC10030468; doi:10.1038/s41467-023-36871-9)
Supplement: Supplementary file 3 — Description of Additional Supplementary Files [file 41467_2023_36871_MOESM3_ESM.pdf]

Description of Additional Supplementary Files

File Name: Supplementary Movie 1
Description: In situ POM showing the **I-II heating** transition

File Name: Supplementary Movie 2
Description: In situ POM showing the **II-I cooling** transition

File Name: Supplementary Movie 3
Description: In situ POM showing the **II-III heating** transition

File Name: Supplementary Movie 4
Description: In situ POM showing the **III-II cooling** transition

File Name: Supplementary Movie 5
Description: In situ POM showing the crack formation during the II-I cooling transition

File Name: Supplementary Movie 6
Description: Video showing the thermosalient motion during the I-II heating transition

File Name: Supplementary Movie 7
Description: GIXD video during the I-II heating transition, images taken at 5 second increments

File Name: Supplementary Movie 8
Description: Movie showing the calculated vibrational mode for the quinoidal molecule at 1581 cm^{-1}

File Name: Supplementary Movie 9
Description: Movie showing the calculated vibrational mode for the quinoidal molecule at 1584 cm^{-1}

File Name: Supplementary Movie 10
Description: Movie showing the calculated vibrational mode for the quinoidal molecule at 1636 cm^{-1}

File Name: Supplementary Movie 11
Description: Movie showing the calculated vibrational mode for the quinoidal molecule at 1878 cm^{-1}

File Name: Supplementary Movie 12
Description: Movie showing the calculated vibrational mode for the quinoidal molecule at 1610 cm^{-1}

File Name: Supplementary Movie 13
Description: GIXD video during the II-III heating transition, images taken at 5 second increments

File Name: Supplementary Movie 14
Description: Movie showing the calculated vibrational mode for the aromatic molecule at 1618 cm^{-1}

File Name: Supplementary Movie 15
Description: Movie showing the calculated vibrational mode for the aromatic molecule at 1635 cm^{-1}
